# Elevated matrix metalloproteinase-9 in patients with systemic sclerosis

**DOI:** 10.1186/ar1454

**Published:** 2004-11-10

**Authors:** Wan-Uk Kim, So-Youn Min, Mi-La Cho, Kyung-Hee Hong, Yong-Joo Shin, Sung-Hwan Park, Chul-Soo Cho

**Affiliations:** 1 Division of Rheumatology, Department of Internal Medicine, Catholic Research Institutes of Medical Science, School of Medicine, The Catholic University of Korea, Seoul, Republic of Korea

**Keywords:** dermal fibroblasts, metalloproteinase-9, skin score, systemic sclerosis

## Abstract

Matrix metalloproteinase-9 (MMP-9) has been implicated in the pathogenesis of cancer, autoimmune disease, and various pathologic conditions characterized by excessive fibrosis. In this study, we investigated the expression of MMP-9 and its clinical significance in systemic sclerosis (SSc). The patients (*n *= 42) with SSc had higher concentrations of MMP-9 and of tissue inhibitor of metalloproteinase-1 (TIMP-1) and a higher ratio of MMP-9 to TIMP-1 in sera than healthy controls (*n *= 32). Serum MMP-9 concentrations were significantly higher in the diffuse type (*n *= 23) than the limited type of SSc (*n *= 19). Serum concentrations of MMP-9 correlated well with the degree of skin involvement, as determined by the Rodnan score and with serum concentrations of transforming growth factor β. Moreover, dermal fibroblasts from patients with SSc produced more MMP-9 than those from healthy controls when they were stimulated with IL-1β, tumor necrosis factor α, or transforming growth factor β. Such an increase in MMP-9 production was partially blocked by treatment with cyclosporin A. In summary, the serum MMP-9 concentrations were elevated in SSc patients and correlated well with skin scores. The increased MMP-9 concentrations may be attributable to overproduction by dermal fibroblasts in SSc. These findings suggest that the enhanced production of MMP-9 may contribute to fibrogenic remodeling during the progression of skin sclerosis in SSc.

## Introduction

Systemic sclerosis (SSc) is a generalized disorder of connective tissue characterized by microvacular damage and excessive fibrosis in the skin and internal organs, including the heart, lungs, and gastrointestinal tract. One of the major hallmarks of the disease is an increased amount of collagen deposits in the affected tissue. The relative proportion of two major types of skin procollagen, types I and III, is higher in SSc lesions than in healthy controls [1,2]. This increase in collagen deposits may be associated with changes in the dermal microvasculature in SSc. In particular, alterations in the structure of the basement membrane, a critical component of the vessel, may lead to changes in the surrounding tissue and to subsequent development of fibrosis in SSc [3]. The finding that the synthesis of type IV collagen, a major collagen type in basement membrane, is disproportionately increased in the dermal fibroblasts and sera of patients with SSc supports this notion [4,5].

The enhanced expression of matrix collagen is presumably associated with abnormal immune responses to collagen in SSc [6-10]. For example, autoantibodies to type IV collagen have been observed in some SSc patients and may be involved in endothelial injury [7,8]. Immunization of mice with autologous type IV collagen leads to the activation of fibroblasts and to fibrosis [9]. Furthermore, type IV collagen activates T cells from patients with SSc [10], suggesting that the selective immunity to type IV collagen may influence the clinical expression of SSc. The excessive production of type IV collagen and subsequent autoimmune T-cell responses to type IV collagen may set off a self-perpetuating cycle in SSc through the interaction between lymphocytes and fibroblasts.

The matrix metalloproteinases (MMPs) are a family of extracellular endopeptidases that selectively degrade the components of various extracellular matrixes. Of these, MMP-9 (92–96 kD gelatinase B), whose substrates include type IV collagen in basement membrane [11], has been thought to be involved in the cellular invasion of the basement membrane by cells involved in arthritis and cancer (e.g. T cells, mononuclear phagocytes, synovial fibroblasts, and metastatic tumor cells) [12-15]. MMP-9 has been associated with chronic inflammatory autoimmune diseases, including rheumatoid arthritis, Sjögren's syndrome, idiopathic uveitis, and systemic lupus erythematosus [16-19]. Moreover, the overexpression of MMP-9 has been reported in various pathologic conditions characterized by excessive fibrosis, including idiopathic pulmonary fibrosis, bronchial asthma, experimental biliary cirrhosis, and chronic pancreatitis [20-23], suggesting that elevated MMP-9 is closely linked to fibrogenic remodeling in target organs. In the present study, we measured the expression of MMP-9 and tissue inhibitor of metalloproteinase-1 (TIMP-1), an inhibitor of MMP-9, in the sera and culture supernatants of dermal fibroblasts from SSc patients and compared them with serum concentrations of transforming growth factor β (TGFβ) and with clinical and laboratory parameters of SSc.

## Materials and methods

### Patients

This study was conducted in accordance with the principles embodied in the Declaration of Helsinki and was approved by the Ethical Committees in the Catholic Research Institutes of Medical Sciences. Before the study, informed consent was obtained from all patients and healthy controls. Forty-two patients (1 man and 41 women), all of whom fulfilled the criteria of the American Rheumatism Association for SSc [24], were studied; their mean age was 43.7 years (range 24–69 years). The mean duration of disease was 80.8 months (range 5–276 months). The comparisons were made with 32 healthy controls (all women) who had no rheumatic disease; their mean age was 44.2 years (range 21–62 years). The ages and sexes of the patient and control groups did not differ significantly.

### Clinical and laboratory evaluation

Clinical and laboratory assessments were done at the time of sampling. The clinical variables were age, sex, disease duration, type of SSc [25], modified Rodan score [26], presence or absence of esophageal involvement on endoscopy and esophageal manometry, interstitial lung disease on chest radioagrapy and/or high-resolution computerized tomography, diffusion capacity (DLCO; diffusion of carbon monoxide in the lung) on the pulmonary function test, arthritis, sicca syndrome, and antibodies to Scl-70 or centromere using ELISA kits (MBL, Nagoya, Japan). Interstitial lung disease was defined as bibasilar interstitial fibrosis on chest radiographs, or, in patients with no abnormalities on chest radiographs, as the presence of alveolitis on high-resolution computerized tomography.

### ELISA for serum MMP-9, TIMP-1, and TGFβ

The total MMP-9 and TIMP-1 concentrations were determined in the serum and the culture supernatant using a commercial ELISA kit (R&D Systems Inc, Minneapolis, MN, USA). In accordance with the manufacturer's recommendations, the aliquots of serum were diluted to a ratio of 1:100 in the assay buffer. The detection limits of the MMP-9 and TIMP-1 kits were 0.15 ng/ml and 0.08 ng/ml, respectively. The MMP-9 assay kit detected pro-MMP-9 and complexes of pro-MMP-9 with TIMP-1 and had no significant cross-reactivity with MMP-1, MMP-2, MMP-3, TIMP-1, or TIMP-2. Again, the TIMP-1 detection kit detected TIMP-1 either free or in complex with some MMPs and showed no cross-reactivity or interference with TIMP-2.

Circulating TGFβ was measured in the same samples using ELISA, as described previously [27]. Briefly, 2 μg/ml of monoclonal antibodies to TGFβ1, β2, and β3 (R&D Systems) were added to 96-well plates (Nunc Inc, Roskilde, Denmark). They were incubated overnight at 4°C and blocked with PBS containing 1% bovine serum albumin and 0.05% Tween 20 for 2 hours at room temperature. A sample (50 μl) of each patient's serum was diluted 1:2 with PBS, acidified with 50 μl of 2.5 M acetic acid and 10 M urea for 10 minutes at room temperature and then was neutralized with 50 μl of 2.7 M NaOH and 1 M HEPES. The patient's sera and the standard recombinant TGFβ (R&D Systems) were then put into 96-well plates and incubated at room temperature for 2 hours. Biotinylated polyclonal antibodies (50 ng/ml) to human TGFβ (R&D Systems) were added and the reaction was allowed to proceed for 2 hours at room temperature. Streptavidin–alkaline phosphatase (Sigma Bioscience, St Louis, MO, USA) diluted 1:2000 with PBS was added, and the reaction was again allowed to proceed for 2 hours. *p*-Nitrophenylphosphate (1 mg/ml) (Sigma Bioscience) dissolved in diethanolamine (Sigma Bioscience) was added to induce a color reaction, and 1 N NaOH (Fisher Scientific, Pittsburgh, PA, USA) was used to stop the reaction. An automated microplate reader (Vmax, Molecular Devices, Palo Alto, CA, USA) was used to measure the optical density at 405 nm. Between each of these steps, the plates were washed four times with PBS containing 0.05% Tween 20. A standard curve was drawn by plotting the optical density versus the log of the recombinant TGFβ concentration. The detection limit for TGFβ was 30 pg/ml.

### Detection of MMP-9 activities by gel zymography

MMP-9 and MMP-2 activities were also tested by gelatin zymography. A 0.5-μl sample of serum diluted in 30 μl of SDS buffer was separated in 10% SDS–PAGE gel polymerized with 1 mg/ml gelatin (Invitrogen Life Technologies, Carlsbad, CA, USA). Culture supernatants of HT1080 cell lines (malignant human fibroblasts) stimulated with 10 μg/ml of concanavalin A were used as a positive control. Gels were washed once for 3 hours in 2.5% Triton X-100 to remove the SDS and once for 30 minutes in the reaction buffer containing 50 mM Tris/HCl, 200 mM NaCl, 10 mM CaCl_2_, and 0.02% (w/v) Brij 35 (pH 7.5). The reaction buffer was changed to a fresh one, and the gels were incubated at 37°C for 24 hours. Gelatinolytic activity was visualized by staining the gels with 0.5% Coomassie brilliant blue and was quantified by densitometry.

### Isolation and culture of dermal fibroblasts

Dermal fibroblasts were obtained from affected skin of two SSc patients and from two healthy controls, as described previously [28]. Fibroblasts were grown from explants in Dulbecco's modified Eagle's medium (DMEM) at 37°C in 5% CO_2_. The cells were then centrifuged at 500 ***g***, resuspended in DMEM supplemented with 10% fetal calf serum (Gibco-BRL, Grand Island, NY, USA), 2 mM glutamine, penicillin (100 U/ml), and streptomycin (100 μg/ml), and plated in 25-cm^2 ^flasks. The cultures were kept at 37°C in 5% CO_2 _and the culture medium was replaced every 3 days. When cells approached confluence, they were detached with trypsin, passed after dilution 1:3 with fresh medium, and recultured until use. Cells were housed in a 37°C humidified incubator with 5% CO_2_. Second- or third-passage cells were used for all experiments. Fibroblasts were seeded in 24-well plates at 5 × 10^4 ^cells per well in serum-free DMEM supplemented with insulin–transferrin–selenium A (ITSA; Gibco BRL). After the cells had been grown in selected medium alone for 12 hours, we added cytokines – IL-1β (10 ng/ml), tumor necrosis factor α (TNF-α) (10 ng/ml), and TGFβ (10 ng/ml) – to stimulate the fibroblasts. After 24 hours of incubation, cell-free media were collected and stored at -20°C until assay. All cultures were set up in triplicate or quadruplicate.

### Statistics

Data are expressed as means ± standard error of the mean (SEM). Numerical data for groups were compared using the Mann–Whitney rank sum test, and data for categories were compared using a chi-square test. Correlation between two variables was tested using Spearman's rank correlation coefficient. *P *values less than 0.05 were considered statistically significant.

## Results

### Elevated serum MMP-9 and TIMP-1 concentrations in SSc patients

**Figure 1:**
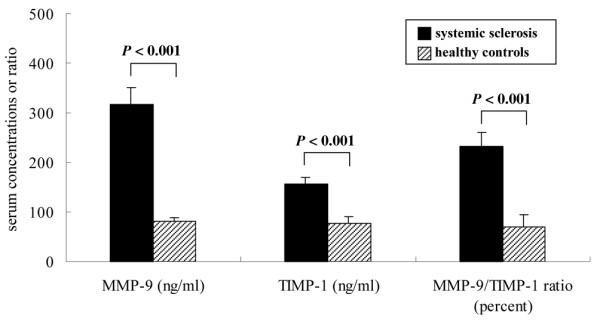
Comparison of serum concentrations of matrix metalloproteinase-9 (MMP-9) and tissue inhibitor of metalloproteinase-1 (TIMP-1) in patients with systemic sclerosis versus healthy controls. Data are presented as means ± standard error of the mean (Mann–Whitney rank sum test).

**Figure 2:**
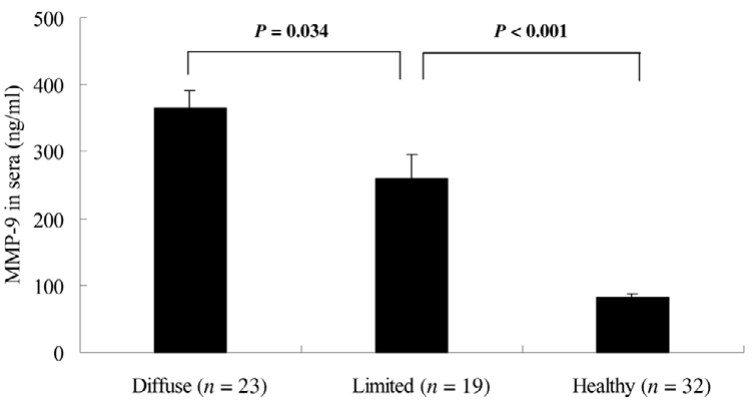
Concentrations of circulating matrix metalloproteinase-9 (MMP-9) in patients with diffuse (*n *= 23) or limited (*n *= 19) systemic sclerosis and in healthy controls (*n *= 32). Data are presented as means ± standard error of the mean (Mann–Whitney rank sum test).

The serum concentrations of MMP-9 were significantly higher in patients with SSc (*n *= 42) than in healthy controls (*n *= 32) (317.6 ± 33.5 ng/ml versus 81.2 ± 6.8 ng/ml, *P *< 0.001) (Fig. 1). The serum concentration of TIMP-1, an inhibitor of MMP-9, was also higher in SSc patients than in healthy controls (157.1 ± 13.2 ng/ml versus 77.7 ± 12.5 ng/ml, *P *< 0.001), but SSc patients had higher MMP-9/TIMP-1 ratios than healthy controls (233.0 ± 27.1 versus 69.5 ± 24.3, *P *< 0.001). There was no correlation between MMP-9 and TIMP-1 concentrations in SSc patients or in healthy controls. SSc patients with the diffuse type (*n *= 23) and had higher concentrations of MMP-9 than those with the limited type (*n *= 19) (364.6 ± 32.4 ng/ml versus 260.0 ± 34.6 ng/ml, *P *= 0.034) (Fig. 2). No significant differences were found between the two groups of patients with regard to age, sex, disease duration, and prednisolone usage or the kinds of immunosuppressive agents being used (e.g. D-penicillamine and cyclosphosphamide) (Table 1).

### MMP-9 activities measured by gel zymography

**Figure 3:**
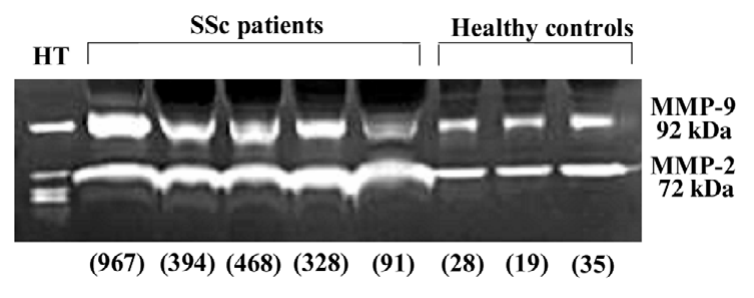
Gelatinase activity of matrix metalloproteinase (MMP)-2 (72 kDa) and MMP-9 (92 kDa) in sera of patients with systemic sclerosis (SSc) and healthy controls. Sera (0.5 μl) from 20 patients with SSc and 10 healthy controls were analyzed for their MMP-2 and MMP-9 activities by gel zymography. As a positive control, supernatants from cultured HT1080 cell lines (HT) stimulated with 10 μg/ml of concanavalin A were used. Numbers in parentheses are MMP-9 concentrations (ng/ml) determined by ELISA. The figure shows representative results for serum samples from the two groups.

We used gel zymography to study sera of 20 SSc patients and 10 healthy controls, all selected unsystematically, to ascertain the serum gelatinase activity of MMP-9. As can be seen in Fig. 3, the 92 kDa band, consistent with the latent form of MMP-9, was detected in the sera of all subjects. The bands in Fig. 3 represent the latent form of MMP-9 (92 kDa, upper band) and the latent form of MMP-2 (72 kDa, lower band). The serum MMP-9 activities of SSc patients were higher than those of healthy controls. Densitometric analysis in sera of 20 SSc patients and 10 healthy controls indicated that the mean MMP-9 activity for SSc patients was 137.2 ± 21.7 densitometry units and for healthy controls, 38.5 ± 4.2 densitometry units (*P *< 0.001). Furthermore, a good linear correlation was found between the densitometry units measured by zymogram and the respective concentrations of MMP-9 measured by immunoassay in the sera of SSc patients (*r *= 0.875 and *P *< 0.001; data not shown). However, the intensity of the 86 kDa band (active MMP-9) was generally weak and was often not measurable.

### Correlation of serum MMP-9 concentrations with skin scores

**Figure 4:**
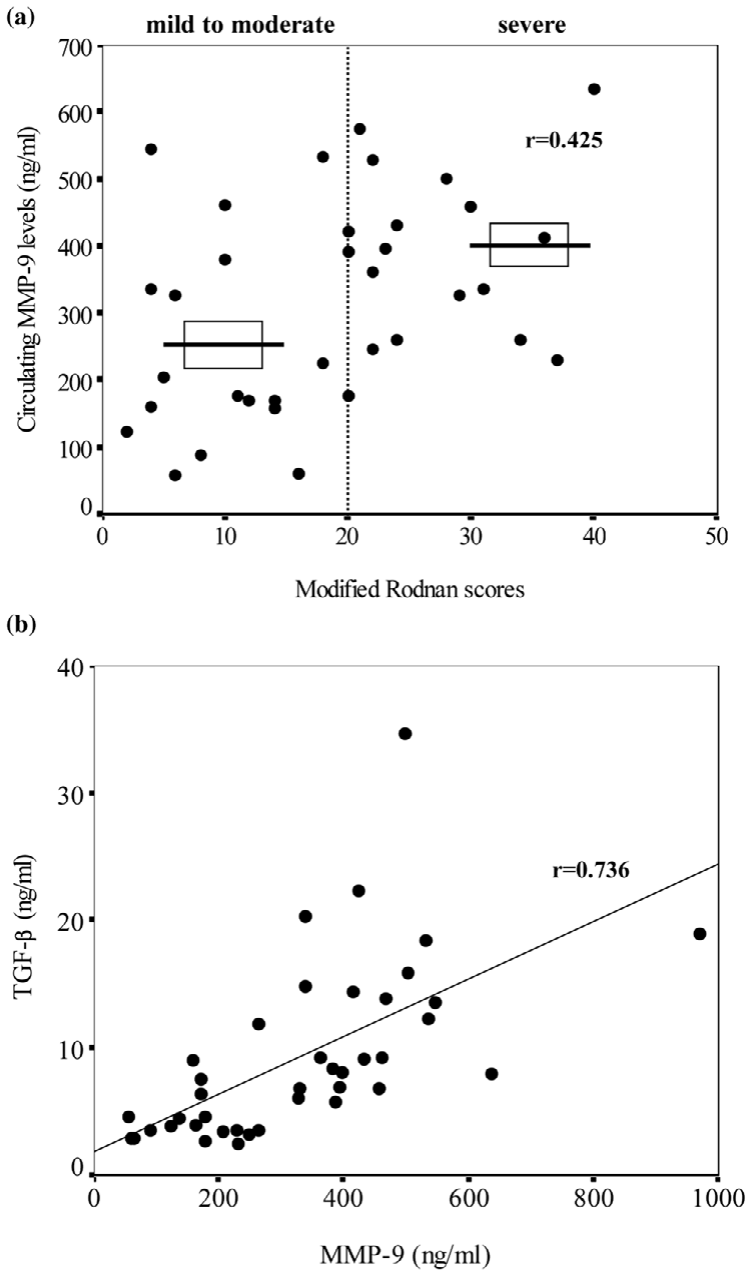
Correlation of circulating matrix metalloproteinase-9 (MMP-9) concentrations with skin fibrosis and with concentrations of transforming growth factor β (TGFβ). **(a) **Correlation of MMP-9 concentrations with skin scores. The extent of skin involvement of systemic sclerosis was determined by modified Rodnan scores. Broken line indicates cutoff value for patients with severe skin involvement (Rodnan score ≥20). Bars represent the mean ± standard error of the mean of MMP-9 in patients with severe versus mild-to-moderate skin involvement (Rodnan score <20). **(b) **Correlation of circulatory MMP-9 concentrations with TGFβ concentrations.

To determine the association of MMP-9 concentrations with a definite clinical manifestation of SSc, we compared the serum MMP-9 concentrations with clinical and laboratory characteristics in patients (*n *= 35) with SSc. The patients with severe skin involvement (*n *= 18), defined by a modified Rodnan score ≥20, had significantly higher concentrations of circulating MMP-9 than those with mild to moderate skin involvement (*n *= 17) (modified Rodnan score <20) (Table 2). Moreover, the serum MMP-9 concentrations correlated well with the Rodnan scores (*n *= 35, *r *= 0.425, *P *= 0.011) and with the serum TGFβ concentrations (*n *= 41, *r *= 0.736, *P *< 0.001) (Fig. 4a,4b). However, a correlation between MMP-9 and TGFβ was not found in the sera from healthy controls (data not shown). There were no differences in the MMP-9 concentrations with respect to age, the presence of esophageal involvement, interstitial lung disease, decrease of diffusion capacity (DLCO < 70%), digital ulcer, arthritis, sicca syndrome, and antibodies to Scl-70 or centromere-B (Table 2).

### MMP-9 production by dermal fibroblasts

**Figure 5:**
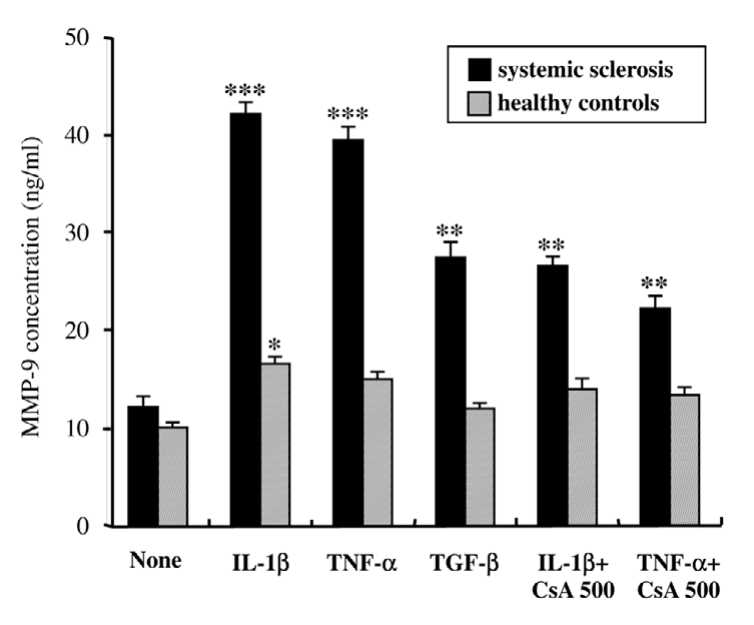
The production of matrix metalloproteinase-9 (MMP-9) and tissue inhibitor of metalloproteinase-1 (TIMP-1) from cultured dermal fibroblasts. Dermal fibroblasts were obtained from affected skin of two patients with systemic sclerosis (SSc) and two healthy controls. Second- or third-passage fibroblasts (5 × 10^4 ^cells) were cultured for 24 hours in Dulbecco's modified Eagle's medium alone and in the presence of IL-1β (10 ng/ml), tumor necrosis factor (TNF)-α (10 ng/ml), transforming growth factor β (TGFβ) (10 ng/ml), IL-1β (10 ng/ml) plus cyclosporin A (CsA) (500 ng/ml), or TNF-α (10 ng/ml) plus CsA (500 ng/ml). The concentrations of MMP-9 in the supernatants were determined by ELISA. Data are expressed as means ± standard error of the mean (SEM) of two independent experiments performed in triplicate using different cell lines. *P < 0.05, **P < 0.01, ***P < 0.001 versus medium alone (Mann–Whitney rank sum test).

The finding that MMP-9 concentrations correlated with skin scores prompted us to investigate the *in vitro *MMP-9 production by dermal fibroblasts from SSc patients. The spontaneous MMP-9 concentrations in the culture supernatants of dermal fibroblasts were not greatly different between SSc patients and healthy controls (Fig. 5). However, stimulation of SSc fibroblasts with IL-1β, TNF-α, or TGFβ strongly increased MMP-9 production relative to the unstimulated concentration, by factors of 3.5, 3.2, and 2.3, respectively, whereas fibroblasts of healthy controls responded weakly to these cytokines (by factors of 1.6, 1.5, and 1.2, respectively). The increase in MMP-9 production by IL-1β and TNF-α appears to be triggered at least in part by a cyclosporin A (CsA)-sensitive pathway, since 500 ng/ml CsA limited MMP-9 production in SSc fibroblasts stimulated with IL-1β or TNF-α to 63% and 57% of original responses, respectively.

## Discussion

We have shown that circulating MMP-9 is higher in patients with SSc than in healthy controls, particularly in the diffuse type of SSc, and correlates well with the extent of skin fibrosis. This finding supports earlier reports that overexpression of MMP-9 is closely linked with various diseases characterized by excessive fibrosis [20-23]. Recent studies support the evidence for a crucial role of MMP-9 in fibrotic diseases. For example, MMP-9-deficient mice exhibit significantly less pulmonary fibrosis in response to bleomycin than their with MMP-9^+/+ ^littermates [29]. In the hepatic fibrosis model infected by *Schistosoma mansoni*, the severity of fibrosis was most closely associated with the increased MMP-9 activity [30]. Similarly, in response to bleomycin, mice deficient in γ-glutamyl transpeptidase showed a reduction in pulmonary fibrosis, in part associated with lower MMP-9 activity in lung tissues [31].

What, then, are the plausible mechanisms by which MMP-9 participates in fibrotic response? One possible explanation comes from the role of MMP-9 in chronic inflammation, resulting in fibrosis. MMP-9 can trigger inflammation directly, by tissue destruction, or indirectly, by generation of an inflammatory signal or recruitment of inflammatory cells [32]. Infiltration of inflammatory cells is closely associated with an abnormal fibrotic response [33]. Moreover, in mice, targeted deletion of MMP-9 attenuated collagen accumulation, which was correlated with decreased infiltration of neutrophils and macrophages in resolving experimental myocardial infarction [34]. In SSc, several proinflammatory cytokines activate fibroblasts to increase MMP-9 production, as depicted in Fig. 5. The overproduced MMP-9 may induce microvascular damage and leakage of substances that further augment endothelial cell damage or fibroblast activation in SSc. This damage may facilitate the movement of inflammatory cells across the basement membrane [11,35], ultimately leading to excessive fibrosis. In this context, type IV collagen autoimmunity, as mentioned in the Introduction, would play an additional role in fibroblast activation through the interaction between T lymphocytes and fibroblasts [9,10]. Such a hypothesis is supported by the findings in SSc patients that microvascular injury precedes fibrosis and that the degree of hypoxia is correlated with skin fibrosis [36,37].

Although the role of TGFβ in SSc remains elusive, several reports have suggested that it may be an ideal candidate as a mediator of skin fibrosis in SSc [38,39]. In the present study, the circulating TGFβ strongly correlated with the MMP-9 concentrations, a finding consistent with the observation that MMP-9 concentrations correlated best with skin scores of SSc. It is known that TGFβ increases the production of MMP-9 in several cell types, possibly through a process requiring protein synthesis that leads to increased statility of MMP-9 mRNA [40,41]. On the other hand, the increased MMP, in turn, is able to cleave latent TGFβ, leading to activation of TGFβ [42], in a process that may constitute a self-perpetuating cycle. If this is the case in SSc patients, MMP-9 may indirectly participate in the fibrotic reaction through the activation of TGFβ, a potent fibrogenic growth factor.

The expression of MMP-9 has been reported to be elevated in the culture medium of alveolar macrophages from patients with idiopathic pulmonary fibrosis or bronchial asthma [20,21,43]. Serum MMP-9 and the MMP-9/TIMP-1 ratio also correlate with the severity of the airway inflammation [44]. In the present study, we did not find any association between serum MMP-9 and the presence or severity of interstitial lung disease, even in a subgroup of SSc patients with diffuse or limited disease (data not shown). The contribution of interstitial lung disease to MMP-9 elevation may be obscured by the stronger effect of skin fibrosis.

The sources of MMP-9 are keratinocytes, monocytes, leukocytes, macrophages, and fibroblasts [12-15]. Fibroblasts from patients with early SSc exhibited higher concentrations of other MMPs (MMP-1 and MMP-3) than fibroblasts from normal individuals [45]. In addition, the finding that MMP-9 correlated best with skin scores prompted us to explore the production of MMP-9 by dermal fibroblasts in SSc patients. This study has shown that SSc fibroblasts produced more MMP-9 after stimulation with IL-1β, TNF-α, and TGFβ than fibroblasts of healthy controls. These findings show that one of the sources for MMP-9 production in SSc is dermal fibroblasts. Moreover, CsA, a calcineurin inhibitor, partially blocked IL-1β-induced or TNF-α-induced MMP-9 production by SSc fibroblasts. This finding suggests that activation of calcineurin and further downstream dephosphorylation of nuclear factor of activated T cells plays a role in the induction of MMP-9 [46] and that CsA may exert its therapeutic effect against SSc [47] by modulating MMP-9 activity.

The findings we report here are in sharp contrast to those in a recently published paper by Kikuchi and colleagues [48], who found decreased concentrations of the active form of MMP-9 in the sera of patients with diffuse SSc. It seems unlikely that this discrepancy is attributable to a difference in the ELISA method (e.g. assay for total MMP-9 in this study versus active MMP-9 in the earlier report), because our patients showed a strong correlation between total MMP-9 and active MMP-9 in the additional test using the ELISA kit (R&D Systems; *r *= 0.745, *P *< 0.001; data not shown). In our study, 33 patients (79%) required corticosteroid plus penicillamine or cyclosphosphamide to control the disease, whereas in the study by Kikuchi and colleagues, only 13 (21%) of 62 patients had been treated with these drugs, suggesting that our patients were in a more active and inflammatory stage of the disease. Given that MMP-9 is abundant in highly inflammatory lesions [32], differences in the stage of disease and clinical features of the patients assessed could account for the opposite results.

Accumulating evidence indicates the importance of TIMP activities in the progression of fibrosis in various pathologic conditions, including asthmatic bronchitis, cirrhosis of the liver, and SSc [49-51]. Moreover, both TIMP1- and TIMP-2 can promote the proliferation of fibroblasts *in vitro *[52,53]. Therefore, it remains to be defined whether the elevated expression of MMP-9 relative to that of TIMP-1 in SSc is directly involved in skin fibrosis or merely reflects biological compensation for excessive fibrosis. Studies of the effect of active MMP-9 or its inhibitor on fibrogenic remodeling in animal models of SSc are needed to clarify this issue.

## Conclusion

Circulating MMP-9 concentrations were elevated in the patients with SSc and correlated best with the skin scores and serum TGFβ concentrations. The production of MMP-9 by dermal fibroblasts of SSc patients was strongly upregulated by stimulation with IL-1β, TNF-α, and TGFβ and such an increase was suppressed by a CsA-sensitive mechanism. Our findings suggest that MMP-9 may play a role in the progression of skin fibrosis in SSc.

## Abbreviations

CsA = cyclosporin A; DMEM = Dulbecco's modified Eagle's medium; ELISA = enzyme-linked immunosorbent assay; IL-1β = interleukin-1β; MMP = matrix metalloproteinase; PBS = phosphate-buffered saline; SSc = systemic sclerosis; TGFβ = transforming growth factor β; TIMP = tissue inhibitor of metalloproteinase; TNF-α = tumor necrosis factor α.

## Competing interests

This work was supported by grants from the Korea Research Foundation Grant (KRF-2002-041-E00107) and the Catholic Research Institutes of Medical Science, Republic of Korea.

## Authors' contributions

W-UK collected the clinical data and analyzed it. S-YM and Y-JS cultured dermal fibroblasts and measured the MMP-9 concentration in the culture supernatant. M-LC performed the gel zymography. K-HH determined the concentrations of MMP-9 and TIMP-1 in the sera. M-LC drafted the manuscript. C-SC designed the study. All authors read and approved the final manuscript.

## Figures and Tables

**Table 1:** Demographics of patients with systemic sclerosis (SSc), and medications being taken

	With diffuse SSc^a ^(*n *= 23)	With limited SSc^a ^(*n *= 19)
Age (y) [mean ± SEM]	42.5 ± 2.8	46.5 ± 2.9
% female	95.7	100
Disease duration (mo.) [mean ± SEM]	72.7 ± 15.7	95.4 ± 17.1
Medications (% of sample)		
Prednisone	82.6	68.4
D-penicillamine	69.6	57.9
Cyclophosphamide	17.4	5.3

^a^*P *≥ 0.05 in a comparison of the two groups (Mann–Whitney rank sum test). SEM, standard error of the mean.

**Table 2:** Association of circulating matrix metalloproteinase (MMP)-9 concentrations with laboratory and clinical variables in patients (*n *= 35) with systemic sclerosis

	MMP-9 (ng/ml)^a^	
	
Variable	Variable present (%)^b^	Variable absent
Modified Rodnan score >20	388 ± 29 (51)*	248 ± 38*
Esophageal involvement^c^	342 ± 33 (63)	304 ± 41
Interstitial lung disease^d^	321 ± 32 (60)	335 ± 41
Decrease of DLCO (<70%)^e^	350 ± 42 (43)	314 ± 34
Digital ulcer	364 ± 32 (43)	260 ± 35
Arthritis	333 ± 58 (14)	333 ± 28
Sicca syndrome	358 ± 40 (31)	320 ± 31
Antibodies to Scl-70^f^	348 ± 36 (37)	323 ± 45
Antibodies to centromere-B^f^	288 ± 34 (37)	370 ± 39

^a^Concentrations are presented as mean ± standard error of the mean. ^b^Percentage of patients in whom the variable was clearly present; all other patients are included in the 'Variable absent' group. ^c^Determined by endoscopy and esophageal manometry. ^d^Evaluated by chest x-ray and/or high-resolution computerized tomography, if necessary. ^e^Abnormal diffusion capacity (DLCO, diffusion of carbon monoxide in the lung) on pulmonary function test was defined as below 70% of that in healthy controls. ^f^Measured by ELISA. *P = 0.006 in a comparison of the two groups (Mann–Whitney rank sum test). All other differences were not significant.
